# Extracorporeal cardiopulmonary resuscitation in adult patients with out-of-hospital cardiac arrest: a retrospective large cohort multicenter study in Japan

**DOI:** 10.1186/s13054-022-03998-y

**Published:** 2022-05-09

**Authors:** Akihiko Inoue, Toru Hifumi, Tetsuya Sakamoto, Hiroshi Okamoto, Jun Kunikata, Hideto Yokoi, Hirotaka Sawano, Yuko Egawa, Shunichi Kato, Kazuhiro Sugiyama, Naofumi Bunya, Takehiko Kasai, Shinichi Ijuin, Shinichi Nakayama, Jun Kanda, Seiya Kanou, Toru Takiguchi, Shoji Yokobori, Hiroaki Takada, Kazushige Inoue, Ichiro Takeuchi, Hiroshi Honzawa, Makoto Kobayashi, Tomohiro Hamagami, Wataru Takayama, Yasuhiro Otomo, Kunihiko Maekawa, Takafumi Shimizu, Satoshi Nara, Michitaka Nasu, Kuniko Takahashi, Yoshihiro Hagiwara, Shigeki Kushimoto, Reo Fukuda, Takayuki Ogura, Shin-ichiro Shiraishi, Ryosuke Zushi, Norio Otani, Migaku Kikuchi, Kazuhiro Watanabe, Takuo Nakagami, Tomohisa Shoko, Nobuya Kitamura, Takayuki Otani, Yoshinori Matsuoka, Makoto Aoki, Masaaki Sakuraya, Hideki Arimoto, Koichiro Homma, Hiromichi Naito, Shunichiro Nakao, Tomoya Okazaki, Yoshio Tahara, Yasuhiro Kuroda, the SAVE-J II study group, Asae Senda, Hajime Suzuki, Atsunori Tanimoto, Kanta Kitagawa, Yoichi Katayama, Nobuaki Igarashi, Masayuki Kawano, Yuji Kuroki, Tadashi Umehara, Yukari Sasaki, Naoki Tominaga, Takuro Hamaguchi, Takuma Sakai, Takeru Abe, Hiroaki Hanafusa, Yuki Yamaoka, Yumi Kakizaki, Shinya Sakato, Shiho Kashiwabara, Takashi Kadoya, Kayo Misumi, Takaomi Kobayashi, Sou Yamada, Masakazu Kobayashi, Naoko Akashi, Masamune Kuno, Jun Maruyama, Hitoshi Kobata, Mitsuhito Soh, Kasumi Shirasaki, Daiki Shiba, Shutaro Isokawa, Masatoshi Uchida, Atsushi Sakurai, Hirotaka Tatsukawa, Marie Nishikawa, Mitsuaki Kojima, Ryohei Kosaki, Takashi Shimazui, Hiroki Kinoshita, Yusuke Sawada, Ryo Yamamoto, Yuya Masuzawa, Kazuki Matsumura, Junya Shimazaki

**Affiliations:** 1 Department of Emergency and Critical Care Medicine, Hyogo Emergency Medical Center, Kobe, Japan; 2 Department of Emergency and Critical Care Medicine, St. Luke’s International Hospital, 9-1 Akashi-cho, Chuo-ku, Tokyo, 104-8560 Japan; 3 Department of Emergency Medicine, Teikyo University School of Medicine, Tokyo, Japan; 4 Department of Critical Care Medicine, St. Luke’s International Hospital, Tokyo, Japan; 5 Clinical Research Support Center, Kagawa University Hospital, Kagawa, Japan; 6 Senri Critical Care Medical Center, Osaka Saiseikai Senri Hospital, Suita, Japan; 7 Advanced Emergency and Critical Care Center, Saitama Red Cross Hospital, Saitama, Japan; 8 Tertiary Emergency Medical Center, Tokyo Metropolitan Bokutoh Hospital, Tokyo, Japan; 9 Department of Emergency Medicine, Sapporo Medical University, Hokkaido, Japan; 10 Department of Emergency and Critical Care Medicine, Nippon Medical School, Tokyo, Japan; 11 Department of Critical Care Medicine and Trauma, National Hospital Organization Disaster Medical Center, Tokyo, Japan; 12 Advanced Critical Care and Emergency Center, Yokohama City University Medical Center, Yokohama, Japan; 13 Tajima Emergency and Critical Care Medical Center, Toyooka Public Hospital, Hyogo, Japan; 14 Trauma and Acute Critical Care Center, Tokyo Medical and Dental University Hospital of Medicine, Tokyo, Japan; 15 Department of Emergency Medicine, Hokkaido University Hospital, Sapporo, Japan; 16 Emergency and Critical Care Medical Center, Teine Keijinkai Hospital, Sapporo, Japan; 17 Department of Emergency and Critical Care Medicine, Urasoe General Hospital, Okinawa, Japan; 18 Department of Emergency Medicine and Critical Care Medicine Tochigi Prefectural Emergency and Critical Care Center, Imperial Gift Foundation Saiseikai, Utsunomiya Hospital, Tochigi, Japan; 19 Division of Emergency and Critical Care Medicine, Tohoku University Graduate School of Medicine, Sendai, Japan; 20 Department of Emergency and Critical Care Medicine, Nippon Medical School Tama Nagayama Hospital, Tokyo, Japan; 21 Department of Emergency Medicine and Critical Care Medicine, Advanced Medical Emergency Department and Critical Care Center, Japan Red Cross Maebashi Hospital, Maebashi, Japan; 22 Department of Emergency and Critical Care Medicine, Aizu Central Hospital, Fukushima, Japan; 23 Emergency Medicine, Osaka Mishima Emergency Critical Care Center, Takatsuki, Japan; 24 Emergency and Critical Care Center, Dokkyo Medical University, Tochigi, Japan; 25 Department of Cardiology, Nihon University Hospital, Tokyo, Japan; 26 Department of Cardiovascular Medicine, Omihachiman Community Medical Center, Shiga, Japan; 27 Department of Emergency and Critical Care Medicine, Tokyo Women’s Medical University Medical Center East, Tokyo, Japan; 28 Department of Emergency and Critical Care Medicine, Kimitsu Chuo Hospital, Chiba, Japan; 29 Department of Emergency Medicine, Hiroshima City Hiroshima Citizens Hospital, Hiroshima, Japan; 30 Department of Emergency Medicine, Kobe City Medical Center General Hospital, Kobe, Japan; 31 Department of Emergency Medicine, Gunma University Graduate School of Medicine, Maebashi, Japan; 32 Department of Emergency and Intensive Care Medicine, JA Hiroshima General Hospital, Hiroshima, Japan; 33 Emergency and Critical Care Medical Center, Osaka City General Hospital, Osaka, Japan; 34 Department of Emergency and Critical Care Medicine, Keio University School of Medicine, Tokyo, Japan; 35 Advanced Emergency and Critical Care Medical Center, Okayama University Hospital, Okayama, Japan; 36 Department of Traumatology and Acute Critical Medicine, Osaka University Graduate School of Medicine, Osaka, Japan; 37 Department of Emergency, Disaster and Critical Care Medicine, Kagawa University Hospital, Kagawa, Japan; 38 Department of Cardiovascular Medicine, National Cerebral and Cardiovascular Center, Suita, Japan

**Keywords:** Real-world data, Extracorporeal cardiopulmonary resuscitation, Out-of-hospital cardiac arrest, Neurological outcome, Survival rate, Complication

## Abstract

**Background:**

The prevalence of extracorporeal cardiopulmonary resuscitation (ECPR) in patients with out-of-hospital cardiac arrest (OHCA) has been increasing rapidly worldwide. However, guidelines or clinical studies do not provide sufficient data on ECPR practice. The aim of this study was to provide real-world data on ECPR for patients with OHCA, including details of complications.

**Methods:**

We did a retrospective database analysis of observational multicenter cohort study in Japan. Adult patients with OHCA of presumed cardiac etiology who received ECPR between 2013 and 2018 were included. The primary outcome was favorable neurological outcome at hospital discharge, defined as a cerebral performance category of 1 or 2.

**Results:**

A total of 1644 patients with OHCA were included in this study. The patient age was 18–93 years (median: 60 years). Shockable rhythm in the initial cardiac rhythm at the scene was 69.4%. The median estimated low flow time was 55 min (interquartile range: 45–66 min). Favorable neurological outcome at hospital discharge was observed in 14.1% of patients, and the rate of survival to hospital discharge was 27.2%. The proportions of favorable neurological outcome at hospital discharge in terms of shockable rhythm, pulseless electrical activity, and asystole were 16.7%, 9.2%, and 3.9%, respectively. Complications were observed during ECPR in 32.7% of patients, and the most common complication was bleeding, with the rates of cannulation site bleeding and other types of hemorrhage at 16.4% and 8.5%, respectively.

**Conclusions:**

In this large cohort, data on the ECPR of 1644 patients with OHCA show that the proportion of favorable neurological outcomes at hospital discharge was 14.1%, survival rate at hospital discharge was 27.2%, and complications were observed during ECPR in 32.7%.

**Supplementary Information:**

The online version contains supplementary material available at 10.1186/s13054-022-03998-y.

## Background

The utilization of extracorporeal membrane oxygenation (ECMO) during conventional cardiopulmonary resuscitation (CPR) is termed extracorporeal CPR (ECPR), and the prevalence of ECPR in patients with out-of-hospital cardiac arrest (OHCA) has been rapidly increasing worldwide [1–9]. In a recent study, promising data regarding the efficacy of ECPR in patients with OHCA younger than 75 years who have refractory ventricular fibrillation (VF) was reported [10], and ECPR will continue to be considered a “life-saving device” around the world. Unlike with conventional CPR [11], there are no practice guidelines on ECPR; therefore, ECPR protocol varies across hospitals.

Initial cardiac rhythm, age, and time (low flow time or no flow time) are commonly used as principal parameters of inclusion criteria in clinical ECPR practice. Several clinical studies on ECPR with small sample sizes have been published [3–9, 12–16]; however, real-world data on ECPR, such as patients with OHCA and non-shockable rhythm, old age, long time from cardiac arrest to initiation of veno-arterial extracorporeal membrane oxygenation (VA-ECMO), have not been thoroughly evaluated. Further, details of ECPR, such as its complications, remain unknown.

Our group designed the retrospective large cohort study known as the Study of Advanced life support for Ventricular fibrillation with Extracorporeal circulation in Japan (SAVE-J II) to provide real-world data on ECPR performed on about 2000 patients [17–19]. The aim of this study was to determine the association between the above-mentioned parameters and outcomes of patients with OHCA who received ECPR.

## Methods

SAVE-J II is a retrospective multicenter registry study with 36 participating institutions in Japan (Additional file 1: Fig. S1). The study was pre-registered at the University Hospital Medical Information Network Clinical Trials Registry, the Japanese clinical trial registry (registration number: UMIN000036490) [18]. This study was approved by the institutional review board of Kagawa University (approval number: 2018-110) and of each participating institution. In all the participating institutions, the requirement for patient consent was waived due to the retrospective nature of this study.

ECPR was defined as resuscitation from cardiac arrest using VA-ECMO. To assess the indications, management, and neurological outcomes, SAVE-J II included consecutive patients’ ≥ 18 years of age who were admitted to the emergency department with OHCA between January 1, 2013 and December 31, 2018 and received ECPR. The current registry excludes patients with OHCA who were transferred to the participating institutions after receiving treatment in another hospital, patients with in‐hospital cardiac arrest (IHCA), and patients who declined to participate through family or other agents. For this study, the following data were collected: patient characteristics, prehospital information, information on hospital arrival, diagnosis and intervention, mechanical support information, time course, body temperature management, intensive care unit (ICU) information, and outcomes [19]. The characteristics of the participating institutions, including inclusion and exclusion criteria and initial resuscitation management, are described in our previous paper [20].

### Study population

We selected patients from SAVE-J II who met the inclusion criterion of initiation of VA-ECMO before ICU admission. The exclusion criteria included non-cardiac conditions such as acute aortic dissection/aortic aneurysm, hypothermia, primary cerebral disorder, infection, drug intoxication, trauma, suffocation, and drowning [17]. Hypothermia was defined as diagnosed by a physician or a body temperature at admission of less than 30 °C [17]. Furthermore, we excluded patients who achieved return of spontaneous circulation (ROSC) on hospital arrival and at ECMO initiation. In addition, patients with unknown outcomes were excluded.

### Data collection

The following patient data were collected from SAVE-J II: age, sex, incidence of witnessed cardiac arrest and bystander CPR, initial cardiac rhythm at the scene and on hospital arrival, cardiac arrest location, use of adrenaline and defibrillation, prehospital airway management, cardiac rhythm before ECMO initiation, treatment related factors, time course, cause of cardiac arrest, ROSC after hospital arrival, and ECMO information. Initial shockable rhythm was defined as VF, pulseless ventricular tachycardia, or rhythm for defibrillation in automated external defibrillator used by emergency medical staff. Location of cardiac arrest at ambulance was defined as patients who developed cardiac arrest after emergency medical staff (EMS) arrival with the presence of spontaneous circulation on initial EMS evaluation. ROSC was defined as at least one minute of continuing confirmation of pulsation. The length of hospital and ICU stay, in-hospital mortality, and neurological outcomes were also collected. Time from call ambulance to arrival was defined as time from emergency medical services call to hospital arrival, time from arrival to ECMO was defined as time from hospital arrival to establishment of ECMO support, time from call ambulance to ECMO was defined as time from emergency medical services call to establishment of ECMO support, estimated low flow time was defined as the time from cardiac arrest to the establishment of ECMO if the location of cardiac arrest was ambulance and the time from calling an ambulance to the establishment of ECMO if the location of cardiac arrest was other than ambulance. Cause of cardiac arrest was classified as acute coronary syndrome [21], arrhythmia [22], myopathy [23], myocarditis [24], other cardiac cause, pulmonary embolism, and other non-cardiac cause. Data on complications during ECPR were also collected. Cannula malposition was defined as cannulation requiring correct the position, or cannulation of wrong vessel such as arterial-arterial and veno-veno cannulation. Unsuccessful cannulation was defined as failure to complete cannulation. Cannulation-related bleeding included cannulation site bleeding and retroperitoneal hemorrhage requiring blood transfusion or surgical intervention/interventional radiology (IVR), and other forms of hemorrhage included intracerebral hemorrhage confirmed on computed tomography (CT), mediastinal hemorrhage, intra-abdominal organ hemorrhage, and gastrointestinal hemorrhage requiring blood transfusion or surgical intervention/IVR.

### Outcome measures

The primary outcome was favorable neurological outcome, evaluated based on the cerebral performance category (CPC) [25] at hospital discharge. A favorable outcome was defined as a CPC of 1 or 2, whereas an unfavorable outcome was defined as a CPC of 3, 4, or 5. The secondary outcomes were survival rate at hospital discharge and complications during ECPR.

### Statistical analysis

Descriptive statistics were used to summarize data on baseline characteristics, outcomes, and complications during ECPR. Categorical variables were counted and presented as proportions. Continuous variables were expressed as medians and interquartile ranges (IQRs). We compared baseline characteristics, outcomes, and complications according to favorable and unfavorable neurological outcomes or according to survival and mortality at hospital discharge using Mann–Whitney U test for continuous variables and Fisher’s exact test or chi-square test for categorical variables, as appropriate. Univariate and multivariable logistic regression analyses were performed for favorable neurological outcome and survival to hospital discharge. We fit logistic regression models with generalized estimating equation to account for patients clustering within the hospital. In the multivariable models, we adjusted for age, sex, witnessed cardiac arrest, bystander CPR, initial cardiac rhythm (shockable rhythm, pulseless electrical activity [PEA], asystole), location of cardiac arrest, and estimated low flow time. Next, we described the baseline characteristics, outcomes, and complications according to initial cardiac rhythm at the scene. We used locally estimated scatterplot smoothing (LOESS) curves with 95% confidence interval to illustrate the relationship between age and the proportion of favorable neurological outcome and survival rate and the relationship between estimated low flow time and the proportion of favorable neurological outcome and survival rate, in all patients and separately for each initial cardiac rhythm. We also used the LOESS curves to illustrate the relationship between age or estimated low flow time and complications. Statistical analyses were performed using R version 3.4.4. and JMP version 12 statistical software (SAS Institute, Cary, NC, USA). Missing data were not replaced or estimated.

## Results

**Fig. 1:**
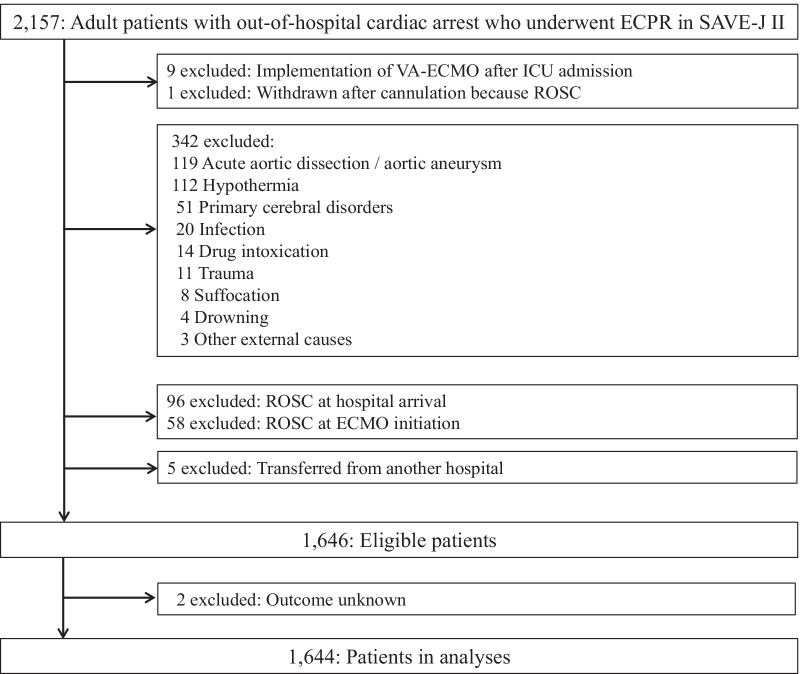
Flowchart of enrollment of study participants. ECPR, extracorporeal cardiopulmonary resuscitation; ECMO,  extracorporeal membrane oxygenation; ICU,  intensive care unit; ROSC,  return of spontaneous circulation

Of the 2157 adult patients with OHCA who received ECPR in SAVE-J II, 1644 were included for analysis (Fig. 1). The distribution of participating institutions and ECPR cases is shown in Additional file 1: Fig S2. Four of the 36 institutions had more than 100 cases of ECPR.

### Characteristics of patients at baseline

Patient age was 18–93 years (median = 60 years). Overall, 84.6% of patients were men, 78.7% had witnessed cardiac arrest, and 58.2% received bystander CPR. The most common location of cardiac arrest was at home (39.9%). The initial cardiac rhythm at the scene was shockable rhythm, PEA, and asystole in 69.4%, 22.6%, and 8.0% of cases, respectively. Regarding prehospital intervention, 64.9% of patients were defibrillated, while 34.5% of patients were administered epinephrine. The median times (and IQRs) from call ambulance to arrival, arrival to ECMO, call ambulance to ECMO, and estimated low flow time were 32 (26–39), 22 (15–32), 56 (47–68), and 55 (45–66) minutes, respectively. ROSC was observed after hospital arrival in 79.0% of patients, and it was observed before ECMO pump on in 17.6% of patients and after ECMO pump on in 82.4% of patients (Table 1). The distributions of age and estimated low flow time are shown in Additional file 1: Figs. S3A and S3B.

**Table 1:** Characteristics of patients at baseline^a^

Variables	Total (*N* = 1644)
Age, years	60 [49–68]
Sex	
Female	254 (15.5)
Male	1390 (84.6)
Comorbidities	1135 (72.6)
Heart disease	420 (25.5)
Location of cardiac arrest	
Home	654 (39.9)
Public place	290 (17.7)
Street	232 (14.2)
Ambulance^b^	183 (11.2)
Workplace	179 (10.9)
Others	101 (6.2)
Initial cardiac rhythm at the scene	
Shockable rhythm	1130 (69.4)
Pulseless electrical activity	368 (22.6)
Asystole	130 (8.0)
Witnessed cardiac arrest	1289 (78.7)
Bystander CPR	945 (58.2)
Prehospital intervention	
Defibrillation	1057 (64.9)
Epinephrine administration	559 (34.5)
Airway management	
No device (bag-mask ventilation)	834 (53.6)
Advanced airway (supraglottic airway)	556 (35.7)
Advanced airway (endotracheal tube)	166 (10.7)
ROSC before hospital arrival	151 (9.3)
Initial cardiac rhythm on hospital arrival	
Shockable rhythm	809 (49.4)
Pulseless electrical activity	498 (30.4)
Asystole	332 (20.3)
Cardiac rhythm at ECMO initiation	
Shockable rhythm	854 (52.4)
Pulseless electrical activity	521 (32.0)
Asystole	254 (15.6)
Time course, minutes	
Time from call ambulance to arrival^c^	32 [26–39]
Time from arrival to ECMO^d^	22 [15–32]
Time from call ambulance to ECMO^e^	56 [47–68]
Estimated low flow time^f^	55 [45–66]
ROSC after hospital arrival	1294 (79.0)
Before ECMO pump on	228 (17.6)
After ECMO pump on	1064 (82.4)
Emergency coronary angiography	1282 (78.0)
Percutaneous coronary intervention	755 (47.5)
Intra-aortic balloon pumping	1060 (64.6)
Cause of cardiac arrest	
Acute coronary syndrome	970 (59.0)
Arrhythmia	232 (14.1)
Myocarditis	19 (1.2)
Myopathy	96 (5.8)
Other cardiac causes	103 (6.3)
Other non-cardiac causes	47 (2.9)
Pulmonary embolism	59 (3.6)
Unknown	117 (7.1)
Cause of death at hospital	
Cardiac arrest as primary cause	1048 (92.0)
Complications	66 (5.8)
Comorbidities	6 (0.5)
Others	19 (1.7)

^a^Data are presented as median [interquartile range] for continuous variables and as *N* (percentage) for categorical variables

CPR, cardiopulmonary resuscitation; ECMO, extracorporeal membrane oxygenation; ROSC, return of spontaneous circulation

^b^Patients who developed cardiac arrest after emergency medical staff (EMS) arrival with the presence of spontaneous circulation on initial EMS evaluation

^c^Call ambulance to arrival time is time from emergency medical services call to hospital arrival time

^d^Arrival to ECMO time is time from hospital arrival to establishment of ECMO support

^e^Call ambulance to ECMO time is time from emergency medical services call to establishment of ECMO support

^f^Estimated low flow time was defined as the time from cardiac arrest to the establishment of ECMO if the location of cardiac arrest was ambulance and the time from calling an ambulance to the establishment of ECMO if the location of cardiac arrest was other than ambulance

Missing data: age = 0, sex = 0, comorbidities = 80, location of cardiac arrest = 5, initial cardiac rhythm at the scene = 16, witnessed cardiac arrest = 6, bystander CPR = 21, defibrillation = 15, epinephrine administration = 23, airway management = 88, ROSC before hospital arrival = 26, initial rhythm on hospital arrival = 5, cardiac rhythm before ECMO initiation = 15, time from call ambulance to arrival = 26, time from arrival to ECMO = 69, time from call ambulance to ECMO = 91, estimated low flow time = 91, ROSC after hospital arrival = 7, time of ROSC = 2, emergency coronary angiography = 1, percutaneous coronary intervention = 55, intra-aortic balloon pumping = 3, cause of cardiac arrest = 1, cause of death at hospital = 58

### Outcomes and complications

Details of the outcomes are shown in Table 2. Favorable neurological outcome at hospital discharge was observed in 14.1% of the 1644 patients. Survival rate at hospital discharge was 27.2%, and in-hospital mortality occurred at a median of 2 (1–4) days. ECPR cases every three months, neurological outcome, and survival to hospital discharge are plotted and shown in Additional file 1: Figs. S4A and S4B. Cannula malposition was observed in 4.9% of patients, and unsuccessful cannulation occurred in 0.7% of patients. Cannulation site bleeding and other types of hemorrhage were observed in 16.4% and 8.5% of patients, respectively. Overall, complications were observed during ECPR in 32.7% of patients.

**Table 2:** Outcome data and complications during extracorporeal cardiopulmonary resuscitation^a^

Variables	Total (*N* = 1644)
Outcomes	
Favorable neurological outcome at hospital discharge	231 (14.1)
Survival to hospital discharge	447 (27.2)
Length of intensive care unit stay, days	3 [1–10]
Length of intensive care unit stay among survivors, days	12 [9–17]
Length of hospital stay, days	3 [1–19]
Length of hospital stay among survivors, days	36 [22–56]
In-hospital mortality, days	2 [1–4]
Complications during ECPR^b^	535 (32.7)
Procedure-related complications^b^	346 (21.2)
Cannula malposition	81 (4.9)
Unsuccessful cannulation	11 (0.7)
Cannulation-related bleeding	268 (16.4)
Others	26 (1.6)
ECMO-related complications	50 (3.1)
Hemorrhage	139 (8.5)
Ischemia	26 (1.6)

^a^Data are presented as median [interquartile range] for continuous variables and as *N* (percentage) for categorical variables

ECPR, extracorporeal cardiopulmonary resuscitation; ECMO, extracorporeal membrane oxygenation

A favorable outcome was defined as a cerebral performance category (CPC) of 1 or 2, whereas an unfavorable outcome was defined as a CPC of 3, 4, or 5

^b^Patients may have more than 1 complication

Missing data: neurological outcome = 0, survival = 0, length of intensive care unit stay = 12, length of intensive care unit stay among survivors = 10, length of hospital stay = 8, length of hospital stay among survivors = 8, in-hospital mortality = 0, complications during ECPR = 6, procedure-related complications = 11, cannula malposition = 7, cannulation failure = 4, cannulation-related bleeding = 5, others = 8, ECMO-related complications = 49, hemorrhage = 5, ischemia = 7

### Comparison and association of characteristics and complications with favorable and unfavorable neurological outcomes and with survival and mortality

Comparisons of patient characteristics and complications according to favorable and unfavorable neurological outcomes and according to survival and mortality are shown in Additional file 1: Tables S1, S2 and S3, respectively. Unadjusted and adjusted associations with favorable neurological outcomes and with survival to hospital discharge are shown in Table 3. Multivariable analysis revealed that age, sex, initial shockable rhythm at the scene, and location of cardiac arrest were significantly associated with both favorable outcome and survival to hospital discharge (*P* < 0.01), and estimated low flow time was significantly associated with survival to hospital discharge (*P* < 0.001).

**Table 3:** Unadjusted and adjusted associations with favorable outcomes (CPC 1 or 2) at hospital discharge and survival to hospital discharge

Variables	Favorable outcome				Survival			
	Univariate analysis		Multivariable analysis^a^		Univariate analysis		Multivariable analysis^a^	
	OR (95% CI)	*P* value	OR (95% CI)	*P* value	OR (95% CI)	*P* value	OR (95% CI)	*P* value
Age	0.98 (0.97–0.98)	< 0.001	0.97 (0.96–0.98)	< 0.001	0.98 (0.97–0.99)	< 0.001	0.98 (0.97–0.99)	< 0.001
Male sex	0.69 (0.49–1.00)	0.049	0.59 (0.41–0.85)	0.005	0.76 (0.57–1.02)	0.071	0.66 (0.53–0.83)	< 0.001
Witnessed cardiac arrest	1.85 (1.26–2.80)	0.001	1.44 (0.90–2.31)	0.126	1.50 (1.13–2.00)	0.004	1.41 (1.06–1.88)	0.018
Bystander CPR	1.95 (1.44–2.67)	< 0.001	1.53 (1.18–1.99)	0.001	1.24 (0.99–1.56)	0.056	0.97 (0.77–1.21)	0.763
Initial cardiac rhythm								
Shockable rhythm	5.02 (2.24–14.32)	< 0.001	4.44 (1.72–11.46)	0.002	3.89 (2.28–7.17)	< 0.001	3.18 (1.67–6.03)	< 0.001
Pulseless electrical activity	2.54 (1.06–7.55)	0.035	1.73 (0.71–4.20)	0.228	1.88 (1.04–3.60)	0.035	1.32 (0.63–2.75)	0.461
Asystole	Reference		Reference		Reference		Reference	
Location of cardiac arrest								
Home	Reference		Reference		Reference		Reference	
Public place	1.70 (1.16–2.49)	0.007	1.75 (1.12–2.73)	0.013	1.63 (1.20–2.20)	0.002	1.63 (1.13–2.34)	0.008
Ambulance^b^	1.86 (1.19–2.86)	0.007	2.28 (1.33–3.92)	0.003	1.52 (1.06–2.17)	0.024	1.77 (1.07–2.93)	0.026
Others	1.11 (0.78–1.57)	0.577	1.13 (0.76–1.68)	0.540	1.12 (0.86–1.46)	0.405	1.11 (0.86–1.44)	0.433
Estimated low flow time^c^	0.99 (0.98–1.00)	0.001	1.00 (0.99–1.00)	0.302	0.98 (0.97–0.98)	< 0.001	0.98 (0.97–0.99)	< 0.001

CPC, cerebral performance category; OR, odds ratio; CI, confidence interval; CPR, cardiopulmonary resuscitation; ECMO, extracorporeal membrane oxygenation

^a^Logistic regression model with generalized estimating equation (GEE) adjusting for age, sex, witnessed cardiac arrest, bystander CPR, initial rhythm (shockable rhythm, pulseless electrical activity, asystole), cardiac arrest (home, public place ambulance, others), and estimated low flow time

^b^Patients who developed cardiac arrest after emergency medical staff (EMS) arrival with the presence of spontaneous circulation on initial EMS evaluation

^c^Estimated low flow time was defined as the time from cardiac arrest to the establishment of ECMO if the location of cardiac arrest was ambulance and the time from calling an ambulance to the establishment of ECMO if the location of cardiac arrest was other than ambulance

### Characteristics, outcomes, and complications according to initial cardiac rhythm, age, and estimated low flow time

**Fig. 2:**
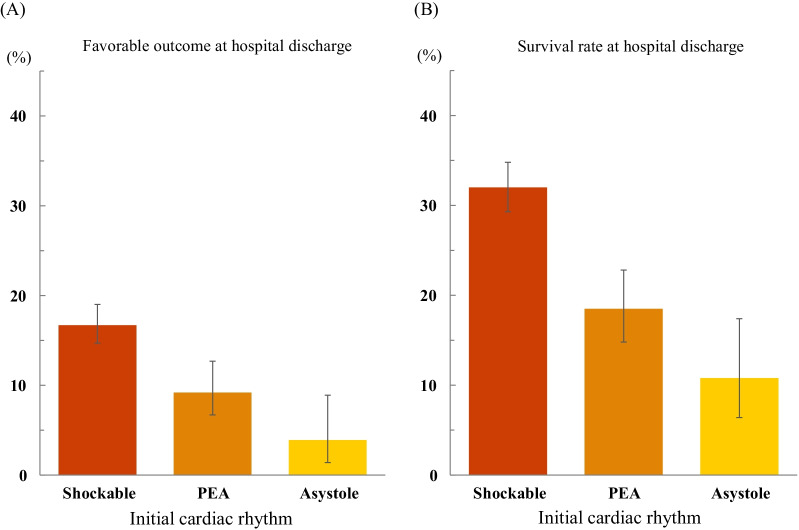
Favorable neurological outcome and survival rate at hospital discharge in initial cardiac rhythm. **A** The proportion of favorable neurological outcome at hospital discharge in initial rhythm at the scene of shockable rhythm, pulseless electrical activity (PEA), and asystole is 16.7%, 9.2%, and 3.9%, respectively. **B** The survival rate at hospital discharge in initial rhythm at the scene of shockable rhythm, PEA, and asystole is 32.0%, 18.5%, and 10.8%, respectively. A favorable outcome is defined as a cerebral performance category (CPC) of 1 or 2, whereas an unfavorable outcome is defined as a CPC of 3, 4, or 5. Data on initial cardiac rhythm at the scene were missing for 16 patients

**Fig. 3:**
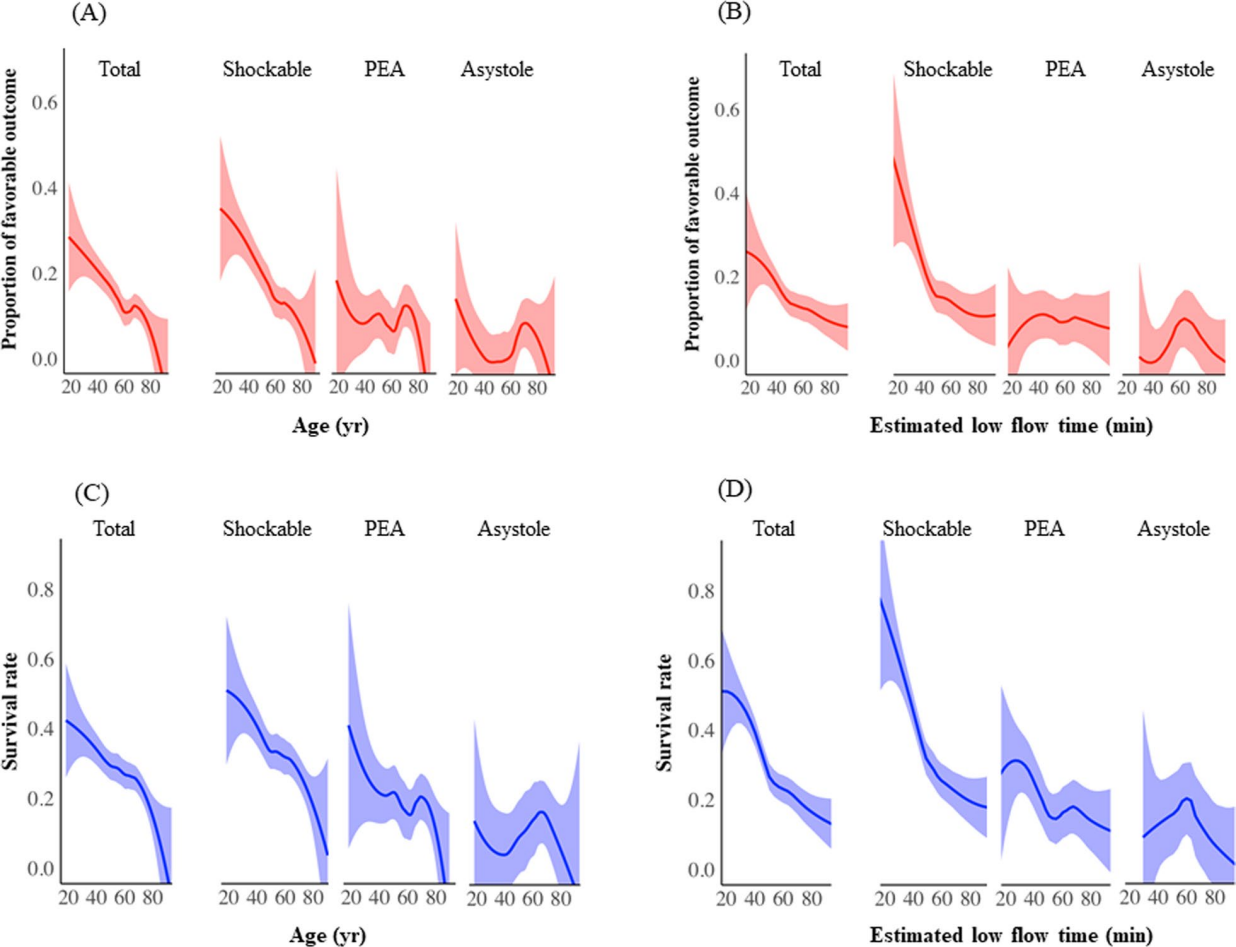
Association between age or estimated low flow time and outcomes. **A**, **C** Association between age and proportion of favorable outcome and survival rate in all patients and for each initial cardiac rhythm (shockable rhythm, PEA, and asystole). **B**, **D** Association between estimated low flow time and proportion of favorable outcome and survival rate in all patients and for each initial cardiac rhythm (shockable rhythm, PEA, and asystole). The bands represent 95% confidence interval (CI). A favorable outcome is defined as a CPC of 1 or 2, whereas an unfavorable outcome is defined as a CPC of 3, 4, or 5

Patient characteristics, outcomes, and complications according to initial cardiac rhythm at the scene are shown in Additional file 1: Tables S4 and S5. The proportions of favorable neurological outcome and survival rate at hospital discharge in terms of shockable rhythm, PEA, and asystole were 16.7%, 9.2%, and 3.9% and 32.0%, 18.5%, and 10.8%, respectively (Fig. 2A, B). The LOESS curve of all patients shows negative relationships between age and the proportion of favorable neurological outcome and survival rate and negative relationships between time and the proportion of favorable neurological outcome and survival rate (Fig. 3). The LOESS curve for each initial cardiac rhythm shows high variability of the proportion of favorable neurological outcome and survival rate (Fig. 3). At a short time interval, patients who had PEA as the initial cardiac rhythm at the scene had a higher survival rate and a lower proportion of favorable neurological outcome than at a longer time interval (Fig. 3B, D). Additional file 1: Fig. S5 shows the LOESS curve of complications. Age and time have weak relationships with complications during ECPR (Additional file 1: Figs S5A and S5E). High age and longtime are associated with high incidence of cannula malposition (Additional file 1: Figs. S5C and S5G) and hemorrhage (Additional file 1: Figs. S5D and S5H).

### ECMO information

Data on ECMO are shown in Additional file 1: Table S6. Vascular access was percutaneous in most patients (98.0%), whereas cut-down was performed in 2.0% of patients. The most common locations for cannulation were emergency department (64.2%) and catheterization room (35.6%). The median (and IQR) size of the ECMO cannula for arteries and veins were 16.5 (15.0–16.5) Fr and 21.0 (19.5–21.0) Fr, respectively. The most common method of cannula removal was the surgical method (59.3%).

## Discussion

This study presents data of 1644 patients with OHCA, of which 69.4% had initial shockable rhythm and 14.1% had favorable neurological outcome at hospital discharge. The proportion of favorable neurological outcome was 16.7%, 9.2%, and 3.9% in patients with shockable rhythm, PEA, and asystole, respectively. The survival rate at hospital discharge was 32.0%, 18.5%, and 10.8% for patients with shockable rhythm, PEA, and asystole, respectively.

This study has several strengths. First, to the best of our knowledge, this study includes the largest cohort of patients with OHCA who received ECPR. Although several large cohort studies of (> 200) patients with OHCA who received ECPR have been reported in France [4], four other European countries [3], Korea [5], and Japan [6, 7, 9], this study included 1.5 times as much participants as the previous study with the largest number (916) of patients [6] and about half the number of patients in a recently published systematic review of ECPR for OHCA [26]. Second, unlike one previous study that included only patients with shockable rhythm [6], this study included patients with all types of cardiac rhythm. Third, this study included four institutions where more than 100 ECPRs were performed. Hence, the data used in this study can be considered a continuous dataset from institutions with low and high volumes of ECPR cases. Finally, this study described complications that were not considered in previous large studies [3–7, 9].

In previous studies that used detailed clinical data of more than 100 patients with OHCA who received ECPR, the proportion of favorable neurological outcome at discharge was 6–39% [3–6, 8, 9, 12–14, 17, 27, 28]. This study included different types of patients, and favorable neurological outcome was observed in 16.7% and 9.2% of patients who had shockable rhythm and PEA, respectively. In this study, the rate of witnessed cardiac arrest was approximately 88%, and bystander CPR was performed in 66% of patients. Further, the median estimated low flow time was 55 min in patients with PEA. The fact that the scatterplot curves of favorable neurological outcome and survival rate are different in terms of estimated low flow time between patients with PEA (i.e., low proportion of favorable neurological outcome despite relatively high survival rate over a short estimated low flow time) and patients with PEA strongly suggests heterogeneity. It is important to carefully determine suitable candidates for ECPR in initial PEA patients. In patients with initial asystole, ECPR may also be considered because, in select populations, the survival rate was greater than 10%.

Age is strongly associated with poor outcomes over time, and it is an independent prognostic factor, as shown with the large dataset used in this study. In previous studies, it was reported that the proportion of favorable neurological outcome in patients over 75 years of age is 1.7–2.9% [6, 7]. It may be necessary to determine cutoffs according to institution, region, and other factors. As with age, the outcomes of estimated low flow time worsened with time. Optimal limited low-flow time, defined as time from CPR initiation to ECPR, was reported by Otani et al. to be 58 min [29], and it was reported in several other studies to be within 60 min [3, 8, 27]. It is difficult to determine optimal limited low-flow time; therefore, it is important to minimize time to ECMO.

Regarding complications associated with ECPR, the most common complication was bleeding, with the rates of cannulation site bleeding and other types of hemorrhage at 16.4% and 8.5%, respectively, but it was reported to be 8–70% in previous studies [1]. Thirty percent of ECPRs were performed in relatively high-volume ECPR centers. This may be because the proportion of obese patients in Japan is lower than those in Western countries [20] and because of the high availability of skilled emergency physicians and acute care surgeons in the emergency departments of high-volume institutions [20]. Since ECPR-related complications are associated with poor outcome [30], it is necessary to shorten the time to ECMO and to initiate ECPR without complications. Training in cannulation technique may be considered in future training systems at high-volume ECPR centers to prevent and control complications.

Broad application of ECPR to patients with OHCA was observed in this study. This seems to be fundamentally associated with the national health insurance system in Japan [31]. Since medical bills are mainly paid by the Japanese government, physicians in charge do not pay attention to unpaid medical bills during ECPR initiation [32]. Moreover, in Japan, trained emergency physicians initiate ECPR with no involvement from cardiovascular surgeons; in contrast, in the USA, cardiovascular surgeons are usually involved throughout the entire ECMO process. Although a recently reported cost-effectiveness study on ECPR for patients with OHCA concluded that ECPR is a robust and economically acceptable resuscitative strategy after considering all parameters [33], it is difficult to establish uniform standard inclusion criteria. It may be possible to expand the indications for ECPR, but any expansion must be balanced by considering factors such as cost, complications, religious views, and local area-specific conditions. The large cohort dataset used in this study contains details of ICU management, CT data, cost, and socioeconomic status; therefore, a statistically confirmed study will provide robust conclusions and further hypotheses on ECPR.

### Limitations

This study has several limitations. First, this was a retrospective study with variation of inclusion criteria in each participating institution; therefore, we reported the inclusion and exclusion criteria of all 36 SAVE-J II hospitals in another paper [20]. Second, there was no control group (i.e., a patient group that did not receive ECPR). Third, data on long-term outcome was not obtained. Fourth, confounders of time course, such as number of ROSC and total ROSC time until ECMO establishment, were not obtained from this dataset. Due to lack of various dataset, estimated low flow time would be different actual low flow time. Fifth, complications were a composite outcome, and specific complications were not assessed.

## Conclusion

In this large cohort, data on the ECPR of 1644 patients with OHCA show that the proportion of favorable neurological outcomes at hospital discharge was 14.1% and survival rate at hospital discharge was 27.2%. Complications were observed during ECPR in 32.7% of patients, and the most common complication was bleeding, with the rates of cannulation site bleeding and other types of hemorrhage at 16.4% and 8.5%, respectively.

## Supplementary Information

**Additional file 1: Fig. S1** Distribution of participating institutions in SAVE-J II. **Fig. S2** Distribution of participating institutions and ECPR From 6 to 135 cases. **Fig. S3** Distribution of age and estimated low flow time. **Fig. S4** ECPR cases and neurological outcome and survival to hospital discharge, 2013–2018. **Fig. S5** Association between age or estimated low flow time and complications. **Table S1.** Baseline characteristics according to neurological outcome at hospital discharge (N = 1644). **Table S2.** Baseline characteristics according to survival to hospital discharge (N = 1644). **Table S3.** Complications during ECPR according to neurological outcomes and survival to hospital discharge (N = 1644). **Table S4.** Baseline characteristics between initial cardiac rhythm at the scene (N = 1628). **Table S5.** Outcome data and complications during ECPR between initial cardiac rhythm at the scene (N = 1628). **Table S6.** ECMO information

## Data Availability

Please contact the author for data requests.

## Abbreviations

CI: Confidence interval
CPC: Cerebral performance category
CPR: Cardiopulmonary resuscitation
CT: Computed tomography
ECMO: Extracorporeal membrane oxygenation
ECPR: Extracorporeal cardiopulmonary resuscitation
EMS: Emergency medical staff
ICU: Intensive care unit
IQRs: Interquartile ranges
IVR: Interventional radiology
LOESS: Locally estimated scatterplot smoothing
OHCA: Out-of-hospital cardiac arrest
OR: Odds ratio
PEA: Pulseless electrical activity
ROSC: Return of spontaneous circulation
VA-ECMO: Veno-arterial extracorporeal membrane oxygenation
VF: Ventricular fibrillation
