# Supplementary material for: Extracorporeal cardiopulmonary resuscitation in adult patients with out-of-hospital cardiac arrest: a retrospective large cohort multicenter study in Japan
Source: Crit Care. 2022 May 9;26:129. doi: 10.1186/s13054-022-03998-y (PMC9088043; doi:10.1186/s13054-022-03998-y)
Supplement: Supplementary file 1 — Additional file 1: Fig. S1 Distribution of participating institutions in SAVE-J II. Fig. S2 Distribution of participating institutions and ECPR From 6 to 135 cases. Fig. S3 Distribution of age and estimated low flow time. Fig. S4 ECPR cases and neurological outcome and survival to hospital discharge, 2013–2018. Fig. S5 Association between age or estimated low flow time and complications. Table S1. Baseline characteristics according to neurological outcome at hospital discharge (N = 1644). Table S2. Baseline characteristics according to survival to hospital discharge (N = 1644). Table S3. Complications during ECPR according to neurological outcomes and survival to hospital discharge (N = 1644). Table S4. Baseline characteristics between initial cardiac rhythm at the scene (N = 1628). Table S5. Outcome data and complications during ECPR between initial cardiac rhythm at the scene (N = 1628). Table S6. ECMO information [file 13054_2022_3998_MOESM1_ESM.docx]

**Additional file 1:**

**Fig. S1 Distribution of participating institutions in SAVE-J II**

A total of 36 institutions were included in SAVE-J II. The annual number of extracorporeal cardiopulmonary resuscitations (ECPRs) according to the number of cases registered in this study is shown.

**Fig. S2 Distribution of participating institutions and ECPR From** **6 to 135 cases**

Between six and 135 ECPRs were performed at the institutions during the study period. More than 100 ECPRs were performed in four of the 36 institutions.

**Fig. S3 Distribution of age and** **estimated low flow time**

The median age was 60 years [interquartile range: 49–68 years], and the median estimated low flow time was 55 [45–66] minutes.

Estimated low flow time was defined as the time from cardiac arrest to the establishment of ECMO if the location of cardiac arrest was ambulance and the time from calling an ambulance to the establishment of ECMO if the location of cardiac arrest was other than ambulance.

**Fig. S4 ECPR cases and** **neurological outcome and survival to hospital discharge, 2013**–**2018**

(A) The light blue portions represent the proportion of favorable outcome, defined as a CPC of 1 or 2, whereas the dark blue portions represent the proportion of unfavorable outcome, defined as a CPC of 3, 4, or 5. (B) The orange portions represent the survival rate, while the gray portions represent the mortality rate.

Extracorporeal cardiopulmonary resuscitation (ECPR) Every three months, 44–90 cases of ECPR were performed, the proportion of favorable outcome was 7.1%–20.3%, and the survival rate was 18.6%–34.4%. The annual number of ECPR cases in 2013 and 2018 was 214 and 333, respectively.

**Fig. S5 Association between age or** **estimated low flow time and complications**

(A) (B) (C) (D) Association between age and incidence of complications

(E) (F) (G) (H) Association between estimated low flow time and incidence of complications

The bands represent 95% CI.

Cannula malposition was defined as cannulation requiring correct the position, or cannulation of wrong vessel such as arterial-arterial and veno-veno cannulation. Unsuccessful cannulation was defined as failure to complete cannulation.

Cannulation-related bleeding includes cannulation site bleeding and retroperitoneal hemorrhage requiring blood transfusion or surgical intervention/interventional radiology (IVR).

Hemorrhage includes intracerebral hemorrhage noted on CT, mediastinal hemorrhage, intra-abdominal organ hemorrhage, and gastrointestinal hemorrhage requiring blood transfusion or surgical intervention/IVR.

**Table S1. Baseline characteristics according to neurological outcome at hospital discharge (N = 1,644)**

**Table S2. Baseline characteristics according to survival to hospital discharge (N = 1,644)**

**Table S3. Complications during ECPR according to** **neurological outcomes and survival to hospital discharge (N= 1,644)**

**Table S4. Baseline characteristics between initial cardiac rhythm at the scene (N = 1,628)**

**Table S5. Outcome data and complications during ECPR between initial cardiac rhythm at the scene (N = 1,628)**

**Table S6.** **ECMO information**


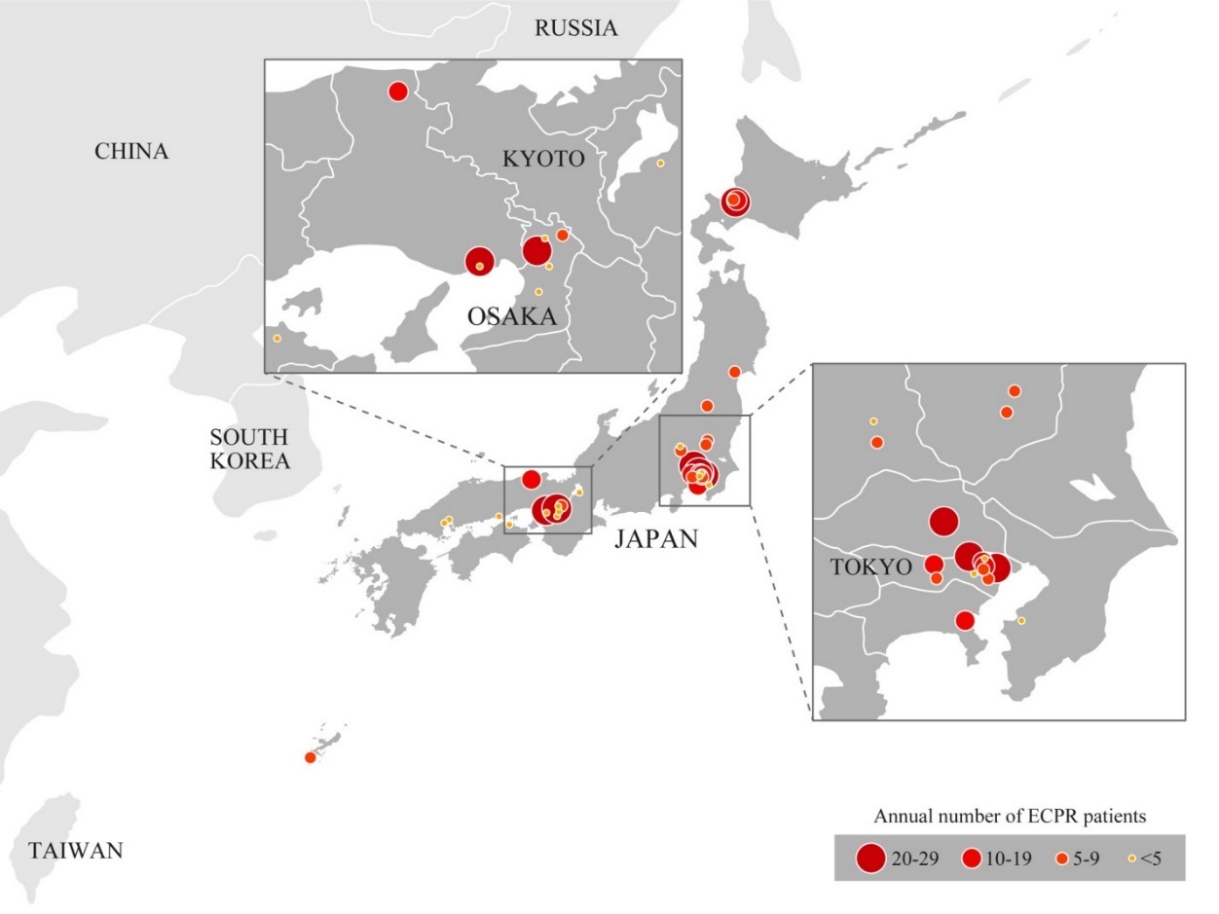


Fig. S1


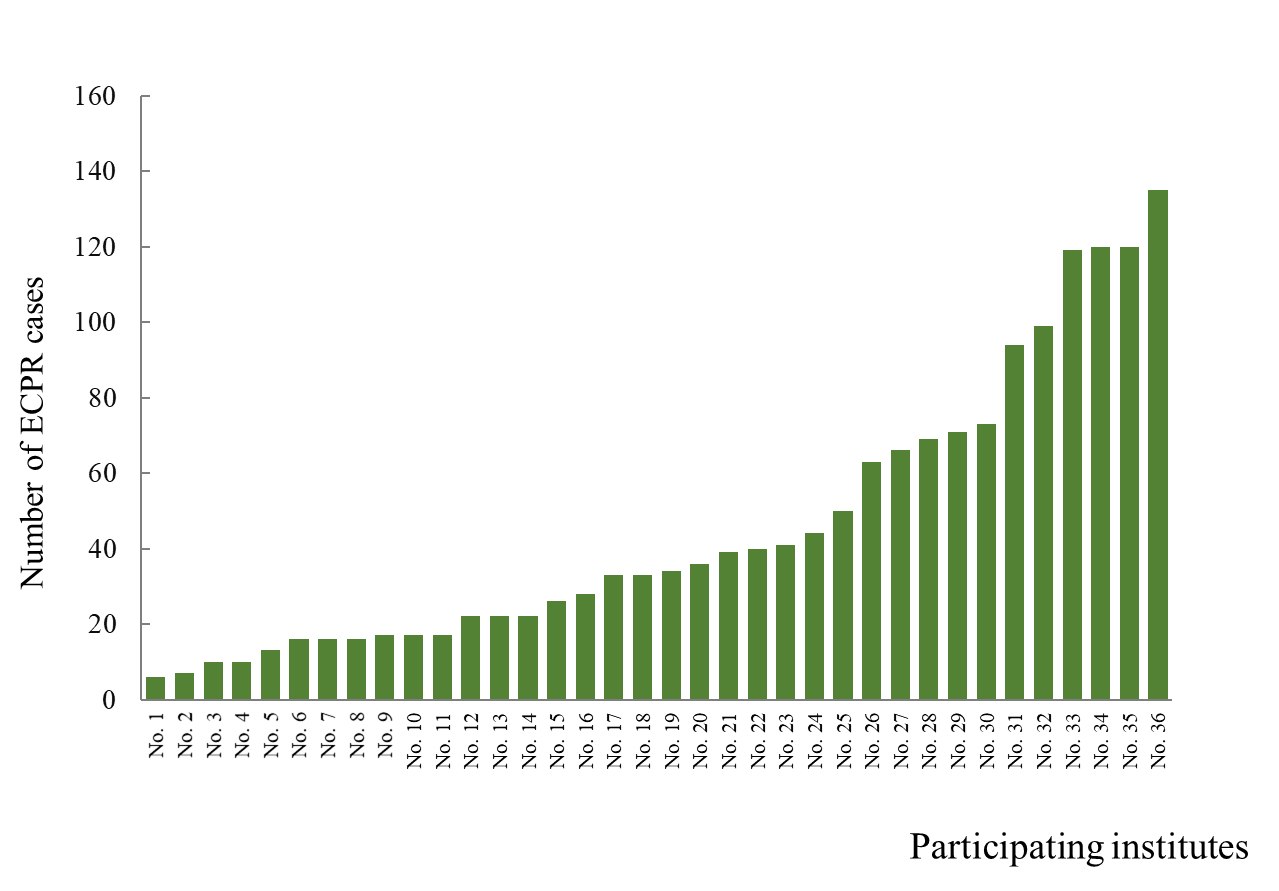


Fig. S2


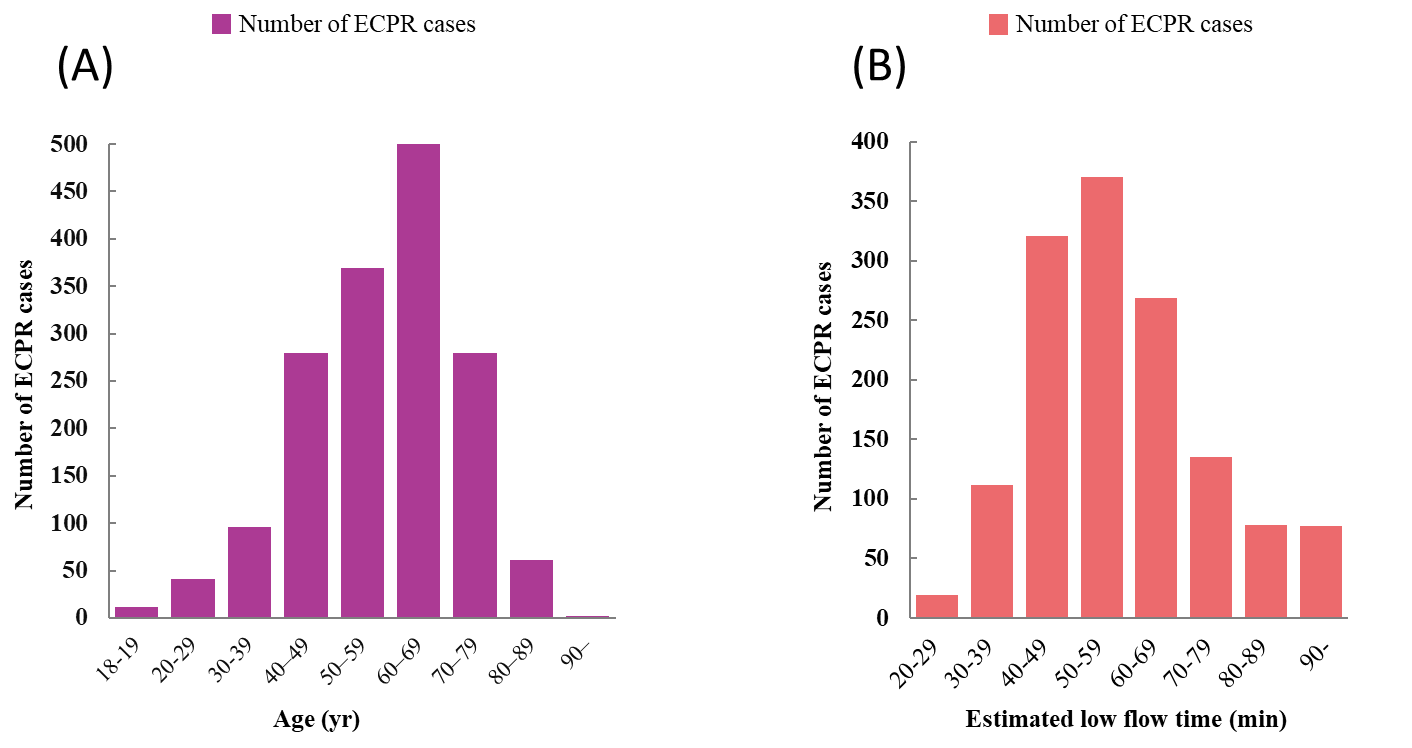


Fig. S3


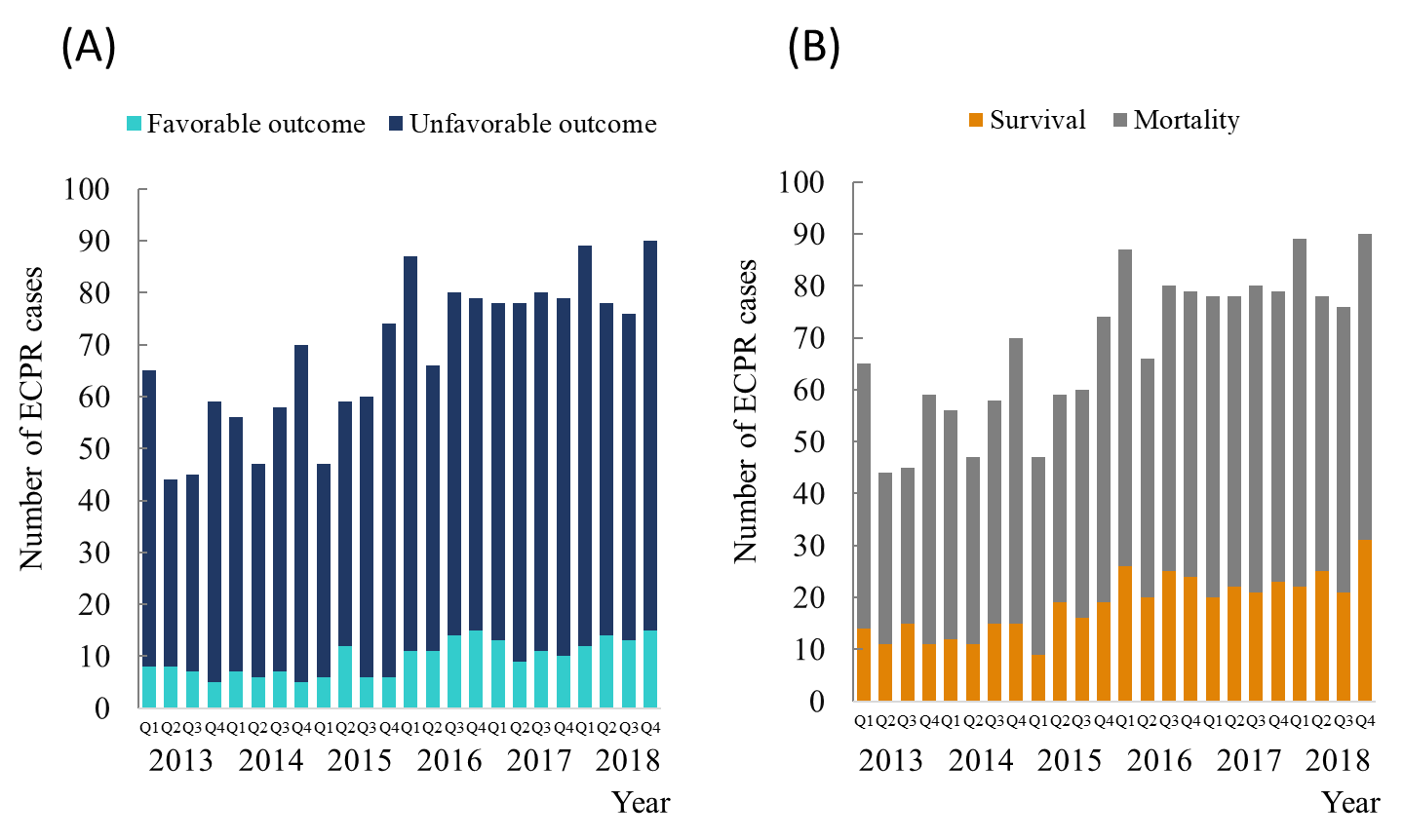


Fig. S4


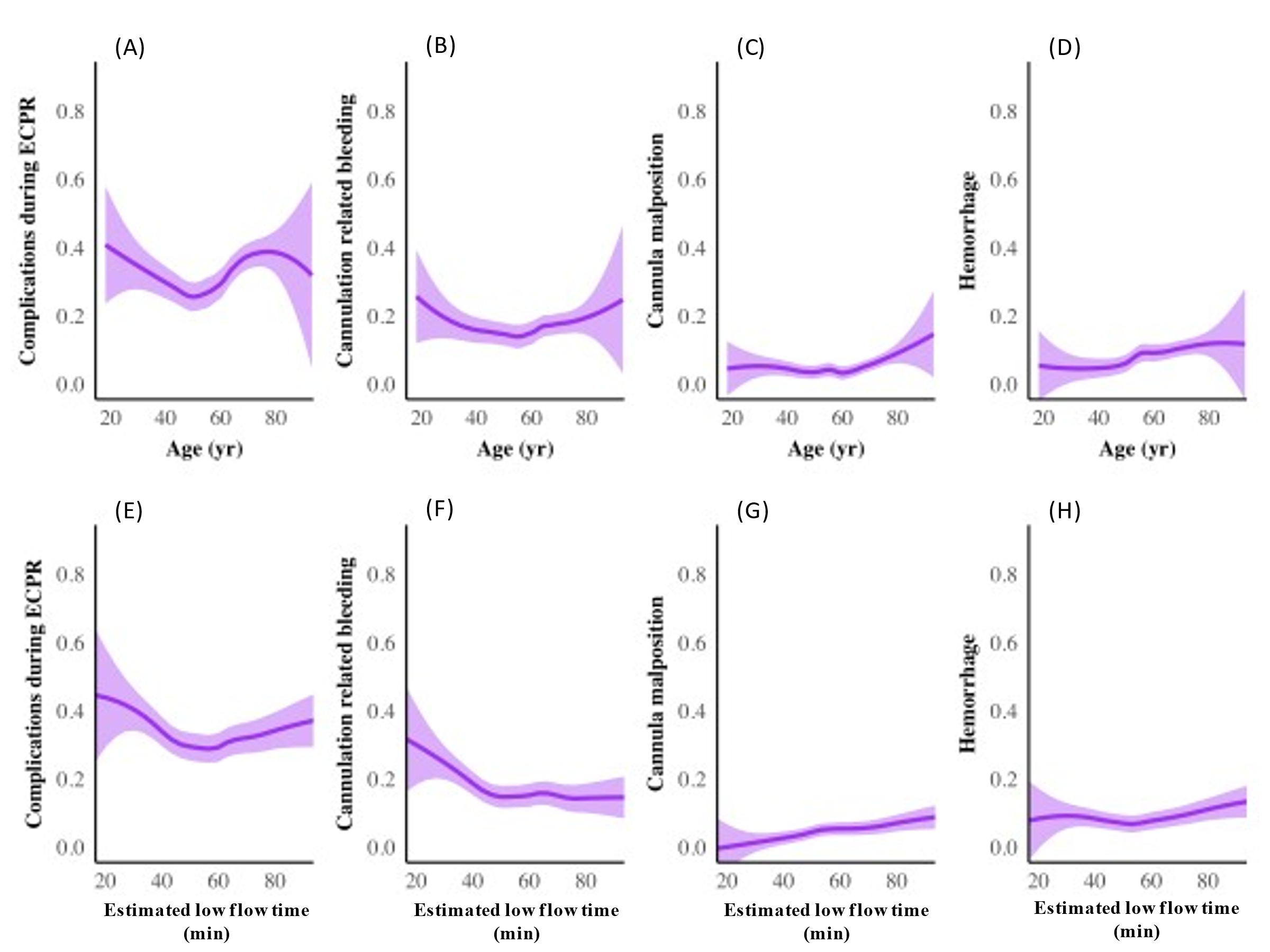


Fig. S5

**Table S1. Baseline characteristics according to neurological outcome at hospital discharge (N =** **1,644)**

| **Variables** | **Favorable outcomes**  **(N = 231)** | **Unfavorable outcomes**  **(N = 1,413)** | **P value** |
| --- | --- | --- | --- |
| Age, years | 55 [45–67] | 61 [50–69] | <0.001 |
| Sex |  |  |  |
| Female | 46 (19.9) | 208 (14.7) | 0.043 |
| Male | 185 (80.1) | 1,205 (85.3) |  |
| Comorbidities | 157 (69.2) | 978 (73.2) | 0.213 |
| Heart disease | 45 (19.5) | 375 (26.5) | 0.023 |
|  |  |  |  |
| Location of cardiac arrest |  |  |  |
| Home | 76 (33.0) | 578 (41.0) | 0.003 |
| Public place | 53 (23.0) | 237 (16.8) |  |
| Ambulance ^#^ | 36 (15.7) | 147 (10.4) |  |
| Street | 23 (10.0) | 209 (14.8) |  |
| Workplace | 31 (13.5) | 148 (10.5) |  |
| Others | 11 (4.8) | 90 (6.4) |  |
| Initial cardiac rhythm at the scene |  |  |  |
| Shockable rhythm | 189 (82.9) | 941 (67.2) | <0.001 |
| Pulseless electrical activity | 34 (14.9) | 334 (23.9) |  |
| Asystole | 5 (2.2) | 125 (8.9) |  |
| Witnessed cardiac arrest | 197 (86.4) | 1,092 (77.5) | 0.002 |
| Bystander CPR | 162 (71.4) | 783 (56.1) | <0.001 |
| Prehospital intervention |  |  |  |
| Defibrillation | 157 (69.2) | 900 (64.2) | 0.146 |
| Epinephrine administration | 62 (27.4) | 497 (35.6) | 0.016 |
| Airway management |  |  |  |
| No device (bag-mask ventilation) | 140 (64.5) | 694 (51.8) | 0.001 |
| Advanced airway (supraglottic airway) | 54 (24.9) | 502 (37.5) |  |
| Advanced airway (endotracheal tube) | 23 (10.6) | 143 (10.7) |  |
| ROSC before hospital arrival | 37 (16.3) | 114 (8.2) | <0.001 |
|  |  |  |  |
| Initial cardiac rhythm on hospital arrival |  |  |  |
| Shockable rhythm | 171 (74.0) | 638 (45.3) | <0.001 |
| Pulseless electrical activity | 52 (22.5) | 446 (31.7) |  |
| Asystole | 8 (3.5) | 324 (23.0) |  |
| Cardiac rhythm at ECMO initiation |  |  |  |
| Shockable rhythm | 169 (73.8) | 685 (48.9) | <0.001 |
| Pulseless electrical activity | 47 (20.5) | 474 (33.9) |  |
| Asystole | 13 (5.7) | 241 (17.2) |  |
|  |  |  |  |
| Time course, minutes |  |  |  |
| Time from call ambulance to arrival ^a^ | 29 [23–37] | 32 [26–39] | 0.001 |
| Time from arrival to ECMO ^b^ | 21 [14–31] | 22 [16–33] | 0.033 |
| Time from call ambulance to ECMO ^c^ | 53 [43–64] | 57 [48–69] | 0.001 |
| Estimated low flow time ^d^ | 51 [41–62] | 55 [46–67] | <0.001 |
| ROSC after hospital arrival | 231 (100) | 1,063 (75.6) | <0.001 |
| Before ECMO pump on | 61 (26.4) | 167 (15.7) | <0.001 |
| After ECMO pump on | 170 (73.6) | 894 (84.3) |  |
| Emergency coronary angiography | 214 (92.6) | 1,068 (75.6) | <0.001 |
| Percutaneous coronary intervention | 120 (54.6) | 635 (46.4) | 0.025 |
| Intraaortic balloon pumping | 173 (74.9) | 887 (62.9) | <0.001 |
| Cause of cardiac arrest |  |  |  |
| Acute coronary syndrome | 140 (60.6) | 830 (58.8) | 0.018 |
| Arrhythmia | 38 (16.5) | 194 (13.7) |  |
| Myocarditis | 4 (1.7) | 15 (1.1) |  |
| Myopathy | 18 (7.8) | 78 (5.5) |  |
| Other cardiac causes | 5 (2.2) | 98 (6.9) |  |
| Other noncardiac causes | 7 (3.0) | 40 (2.8) |  |
| Pulmonary embolism | 11 (4.8) | 48 (3.4) |  |
| Unknown | 8 (3.5) | 109 (7.7) |  |

Data are presented as median [interquartile range] for continuous variables and as *N* (percentage) for categorical variables.

CPR = cardiopulmonary resuscitation, ROSC = return of spontaneous circulation, ECMO = extracorporeal membrane oxygenation

^#^ Patients who developed cardiac arrest after emergency medical staff (EMS) arrival with the presence of spontaneous circulation on initial EMS evaluation.

^a^ Call ambulance to arrival time is time from emergency medical services call to hospital arrival time.

^b^ Arrival to ECMO time is time from hospital arrival to establishment of ECMO support.

^c^ Call ambulance to ECMO time is time from emergency medical services call to establishment of ECMO support.

^d^ Estimated low flow time was defined as the time from cardiac arrest to the establishment of ECMO if the location of cardiac arrest was ambulance and the time from calling an ambulance to the establishment of ECMO if the location of cardiac arrest was other than ambulance

**Table S2. Baseline characteristics according to survival to hospital discharge (N = 1,644)**

| **Variables** | **Survival**  **(N = 447)** | **Death**  **(N = 1,197)** | **P value** |
| --- | --- | --- | --- |
| Age, years | 58 [46–66] | 61 [50–69] | <0.001 |
| Sex |  |  |  |
| Female | 81 (18.1) | 173 (14.5) | 0.067 |
| Male | 366 (81.9) | 1,024 (85.6) |  |
| Comorbidities | 314 (71.5) | 821 (73.0) | 0.563 |
| Heart disease | 95 (21.3) | 325 (27.2) | 0.015 |
|  |  |  |  |
| Location of cardiac arrest |  |  |  |
| Home | 156 (35.0) | 498 (41.7) | 0.014 |
| Public place | 98 (22.0) | 192 (16.1) |  |
| Ambulance ^#^ | 59 (13.2) | 124 (10.4) |  |
| Street | 55 (12.3) | 177 (14.8) |  |
| Workplace | 52 (11.7) | 127 (10.7) |  |
| Others | 26 (5.8) | 75 (6.3) |  |
| Initial cardiac rhythm at the scene |  |  |  |
| Shockable rhythm | 361 (81.5) | 769 (64.9) | <0.001 |
| Pulseless electrical activity | 68 (15.4) | 300 (25.3) |  |
| Asystole | 14 (3.2) | 116 (9.8) |  |
| Witnessed cardiac arrest | 370 (83.3) | 919 (77.0) | 0.005 |
| Bystander CPR | 273 (62.1) | 672 (56.8) | 0.057 |
| Prehospital intervention |  |  |  |
| Defibrillation | 321 (72.8) | 736 (62.0) | <0.001 |
| Epinephrine administration | 138 (31.4) | 421 (35.6) | 0.115 |
| Airway management |  |  |  |
| No device (bag-mask ventilation) | 245 (57.7) | 589 (52.1) | 0.130 |
| Advanced airway (supraglottic airway) | 141 (33.2) | 415 (36.7) |  |
| Advanced airway (endotracheal tube) | 39 (9.2) | 127 (11.2) |  |
| ROSC before hospital arrival | 65 (14.9) | 86 (7.3) | <0.001 |
|  |  |  |  |
| Initial cardiac rhythm on hospital arrival |  |  |  |
| Shockable rhythm | 304 (68.0) | 505 (42.4) | <0.001 |
| Pulseless electrical activity | 111 (24.8) | 387 (32.5) |  |
| Asystole | 32 (7.2) | 300 (25.2) |  |
| Cardiac rhythm at ECMO initiation |  |  |  |
| Shockable rhythm | 311 (69.9) | 543 (45.9) | <0.001 |
| Pulseless electrical activity | 108 (24.3) | 413 (34.9) |  |
| Asystole | 26 (5.8) | 228 (19.3) |  |
|  |  |  |  |
| Time course, minutes |  |  |  |
| Time from call ambulance to arrival ^a^ | 29 [24–36] | 33 [26–40] | <0.001 |
| Time from arrival to ECMO ^b^ | 20 [14–29] | 23 [16–34] | <0.001 |
| Time from call ambulance to ECMO ^c^ | 52 [42–63] | 58 [49–70] | <0.001 |
| Estimated low flow time ^d^ | 50 [40–60] | 56 [47–68] | <0.001 |
| ROSC after hospital arrival | 447 (100) | 847 (71.2) | <0.001 |
| Before ECMO pump on | 104 (23.3) | 124 (14.7) | <0.001 |
| After ECMO pump on | 343 (76.7) | 721 (85.3) |  |
| Emergency coronary angiography | 414 (92.6) | 868 (72.6) | <0.001 |
| Percutaneous coronary intervention | 240 (55.7) | 515 (44.5) | <0.001 |
| Intraaortic balloon pumping | 352 (78.9) | 708 (59.3) | <0.001 |
| Cause of cardiac arrest |  |  |  |
| Acute coronary syndrome | 279 (62.4) | 691 (57.8) | <0.001 |
| Arrhythmia | 75 (16.8) | 157 (13.1) |  |
| Myocarditis | 7 (1.6) | 12 (1.0) |  |
| Myopathy | 29 (6.5) | 67 (5.6) |  |
| Other cardiac causes | 14 (3.1) | 89 (7.4) |  |
| Other noncardiac causes | 11 (2.5) | 36 (3.0) |  |
| Pulmonary embolism | 19 (4.3) | 40 (3.3) |  |
| Unknown | 13 (2.9) | 104 (8.7) |  |

Data are presented as median [interquartile range] for continuous variables and as *N* (percentage) for categorical variables.

CPR = cardiopulmonary resuscitation, ROSC = return of spontaneous circulation, ECMO = extracorporeal membrane oxygenation

^#^ Patients who developed cardiac arrest after emergency medical staff (EMS) arrival with the presence of spontaneous circulation on initial EMS evaluation.

^a^ Call ambulance to arrival time is time from emergency medical services call to hospital arrival time.

^b^ Arrival to ECMO time is time from hospital arrival to establishment of ECMO support.

^c^ Call ambulance to ECMO time is time from emergency medical services call to establishment of ECMO support.

^d^ Estimated low flow time was defined as the time from cardiac arrest to the establishment of ECMO if the location of cardiac arrest was ambulance and the time from calling an ambulance to the establishment of ECMO if the location of cardiac arrest was other than ambulance

**Table S3. Complications during ECPR according to neurological outcomes and survival to hospital discharge (N = 1,644)**

| **Variables** |  | **Favorable outcomes**  **(N = 231)** | **Unfavorable outcomes**  **(N = 1,413)** | **P value** |  | **Survival**  **(N = 447)** | **Death**  **(N = 1,197)** | **P value** |
| --- | --- | --- | --- | --- | --- | --- | --- | --- |
| Complications during ECPR ^a^ |  | 80 (34.8) | 455 (32.3) | 0.460 |  | 169 (37.9) | 366 (30.7) | 0.006 |
| Procedure-related complications ^a^ |  | 47 (20.5) | 299 (21.3) | 0.791 |  | 113 (25.5) | 233 (19.6) | 0.010 |
| Cannula malposition ^b^ |  | 8 (3.5) | 73 (5.2) | 0.268 |  | 16 (3.6) | 65 (5.5) | 0.123 |
| Unsuccessful cannulation ^c^ |  | 0 | 11 (0.8) | 0.380 |  | 0 (0) | 11 (0.9) | 0.042 |
| Cannulation-related bleeding ^d^ |  | 39 (16.9) | 229 (16.3) | 0.814 |  | 100 (22.4) | 168 (14.1) | <0.001 |
| Others |  | 3 (1.3) | 23 (1.6) | 1.000 |  | 5 (1.1) | 21 (1.8) | 0.505 |
| ECMO-related complications ^e^ |  | 9 (3.9) | 41 (3.0) | 0.416 |  | 16 (3.6) | 34 (3.0) | 0.522 |
| Hemorrhage ^f^ |  | 14 (6.1) | 125 (8.9) | 0.160 |  | 38 (8.5) | 101 (8.5) | 0.972 |
| Ischemia ^g^ |  | 7 (3.0) | 19 (1.4) | 0.081 |  | 11 (2.5) | 15 (1.3) | 0.117 |

Data are presented as median [interquartile range] for continuous variables and as *N* (percentage) for categorical variables.

ECPR = extracorporeal cardiopulmonary resuscitation, ECMO = extracorporeal membrane oxygenation

A favorable outcome was defined as a CPC of 1 or 2, whereas an unfavorable outcome was defined as a CPC of 3, 4, or 5.

^a^ Patients may have more than 1 complication.

^b^ Cannulation requiring correct the position, or cannulation of wrong vessel such as arterial-arterial and veno-veno cannulation

^c^ Failure to complete cannulation

^d^ Including cannulation site bleeding and retroperitoneal hemorrhage requiring blood transfusion or surgical intervention/interventional radiology (IVR)

^e^ Equipment malfunction, hemolysis, and other complications related to ECMO

^f^ Including intracerebral hemorrhage noted on computed tomography (CT), mediastinal hemorrhage, intraabdominal organ hemorrhage, and gastrointestinal hemorrhage requiring blood transfusion or surgical intervention/IVR

^g^ Including cerebral infarction and mesenteric ischemia

**Table S4. Baseline characteristics between initial cardiac rhythm at the scene (N =** **1,628)**

| **Variables** | **Shockable rhythm**  **(N = 1,130)** | **PEA**  **(N = 368)** | **Asystole**  **(N = 130)** |
| --- | --- | --- | --- |
| Age, years | 59 [49–68] | 62 [50–69] | 61 [49–68] |
| Sex |  |  |  |
| Female | 140 (12.4) | 90 (24.5) | 19 (14.6) |
| Male | 990 (87.6) | 278 (75.5) | 111 (85.4) |
| Comorbidities | 785 (72.6) | 259 (74.6) | 76 (63.3) |
| Heart disease | 311 (27.5) | 84 (22.8) | 21 (16.2) |
|  |  |  |  |
| Location of cardiac arrest |  |  |  |
| Home | 465 (41.3) | 136 (37.1) | 49 (38.0) |
| Public place | 218 (19.3) | 42 (11.4) | 30 (23.3) |
| Street | 171 (15.2) | 40 (10.9) | 19 (14.7) |
| Ambulance ^#^ | 65 (5.8) | 103 (28.1) | 10 (7.8) |
| Workplace | 138 (12.2) | 32 (8.7) | 7 (5.4) |
| Others | 70 (6.2) | 14 (3.8) | 14 (10.9) |
| Witnessed cardiac arrest | 873 (77.3) | 324 (88.3) | 82 (63.1) |
| Bystander CPR | 631 (56.5) | 239 (65.5) | 68 (52.3) |
| Prehospital intervention |  |  |  |
| Defibrillation | 919 (81.8) | 80 (21.9) | 54 (41.5) |
| Epinephrine administration | 407 (36.4) | 109 (30.0) | 42 (32.3) |
| Airway management |  |  |  |
| No device (bag-mask ventilation) | 567 (53.1) | 209 (58.9) | 53 (43.1) |
| Advanced airway (supraglottic airway) | 379 (35.5) | 118 (33.2) | 55 (44.7) |
| Advanced airway (endotracheal tube) | 121 (11.3) | 28 (7.9) | 15 (12.2) |
| ROSC before hospital arrival | 89 (8.0) | 47 (12.8) | 13 (10.1) |
|  |  |  |  |
| Initial cardiac rhythm on hospital arrival |  |  |  |
| Shockable rhythm | 703 (62.4) | 67 (18.3) | 35 (26.9) |
| Pulseless electrical activity | 234 (20.8) | 223 (60.9) | 35 (26.9) |
| Asystole | 190 (16.9) | 76 (20.8) | 60 (46.2) |
| Cardiac rhythm at ECMO initiation |  |  |  |
| Shockable rhythm | 720 (64.3) | 85 (23.4) | 44 (34.1) |
| Pulseless electrical activity | 248 (22.1) | 218 (59.9) | 48 (37.2) |
| Asystole | 152 (13.6) | 61 (16.8) | 37 (28.7) |
|  |  |  |  |
| Time course, minutes |  |  |  |
| Time from call ambulance to arrival ^a^ | 31 [25–37] | 33 [27–40] | 34 [28–44] |
| Time from arrival to ECMO ^b^ | 20 [15–30] | 25 [18–37] | 27 [19–44] |
| Time from call ambulance to ECMO ^c^ | 54 [46–65] | 60 [51–73] | 63 [53–78] |
| Estimated low flow time ^d^ | 54 [45–64] | 55 [45–69] | 62 [53–76] |
| ROSC after hospital arrival | 904 (80.5) | 291 (79.1) | 87 (66.9) |
| Before ECMO pump on | 132 (14.6) | 71 (24.4) | 21 (24.1) |
| After ECMO pump on | 770 (85.4) | 220 (75.6) | 66 (75.9) |
| Emergency coronary angiography | 935 (82.8) | 254 (69.0) | 84 (64.6) |
| Percutaneous coronary intervention | 560 (50.8) | 144 (41.9) | 45 (35.7) |
| Intraaortic balloon pumping | 781 (69.2) | 202 (55.2) | 68 (52.3) |
| Cause of cardiac arrest |  |  |  |
| Acute coronary syndrome | 706 (62.5) | 195 (53.0) | 63 (48.5) |
| Arrhythmia | 186 (16.5) | 26 (7.1) | 19 (14.6) |
| Myocarditis | 13 (1.2) | 3 (0.8) | 3 (2.3) |
| Myopathy | 80 (7.1) | 10 (2.7) | 5 (3.9) |
| Other cardiac causes | 56 (5.0) | 32 (8.7) | 12 (9.2) |
| Other noncardiac causes | 20 (1.8) | 20 (5.4) | 7 (5.4) |
| Pulmonary embolism | 3 (0.3) | 48 (13.0) | 7 (5.4) |
| Unknown | 65 (5.8) | 34 (9.2) | 14 (10.8) |
| Cause of death at hospital |  |  |  |
| Primary cause of cardiac arrest as primary cause | 664 (91.1) | 265 (92.7) | 108 (96.4) |
| Complications | 48 (6.6) | 17 (5.9) | 0 (0) |
| Comorbidities | 6 (0.8) | 0 (0) | 0 (0) |
| Others | 11 (1.5) | 4 (1.4) | 4 (3.6) |

Data are presented as median [interquartile range] for continuous variables and as *N* (percentage) for categorical variables.

CPR = cardiopulmonary resuscitation, ROSC = return of spontaneous circulation, ECMO = extracorporeal membrane oxygenation

^#^ Patients who developed cardiac arrest after emergency medical staff (EMS) arrival with the presence of spontaneous circulation on initial EMS evaluation.

^a^ Call ambulance to arrival time is time from emergency medical services call to hospital arrival time.

^b^ Arrival to ECMO time is time from hospital arrival to establishment of ECMO support.

^c^ Call ambulance to ECMO time is time from emergency medical services call to establishment of ECMO support.

^d^ Estimated low flow time was defined as the time from cardiac arrest to the establishment of ECMO if the location of cardiac arrest was ambulance and the time from calling an ambulance to the establishment of ECMO if the location of cardiac arrest was other than ambulance

Missing data on initial cardiac rhythm at the scene = 16

**Table S5. Outcome data and complications during ECPR between initial cardiac rhythm at the scene (N = 1,628)**

| **Variables** | **Shockable rhythm**  **(N = 1,130)** | **PEA**  **(N = 368)** | **Asystole**  **(N = 130)** |
| --- | --- | --- | --- |
| Outcomes |  |  |  |
| Favorable outcome at hospital discharge | 189 (16.7) | 34 (9.2) | 5 (3.9) |
| Survival rate | 361 (32.0) | 68 (18.5) | 14 (10.8) |
| Length of intensive care unit stay, days | 4 [1–11] | 3 [1–9] | 2 [1–7] |
| Length of intensive care unit stay among survivors, days | 12 [9–17] | 14 [9–22] | 15 [9–20] |
| Length of hospital stay, days | 4 [2–23] | 3 [1–14] | 2 [1–9] |
| Length of hospital stay among survivors, days | 35 [22–54] | 38 [21–74] | 41 [28–47] |
| In-hospital mortality, days | 2 [1–4] | 2 [1–5] | 2 [1–4] |
|  |  |  |  |
| Complications during ECPR ^a^ | 362 (32.2) | 124 (33.8) | 47 (36.2) |
| Procedure-related complications ^a^ | 237 (21.1) | 76 (20.9) | 32 (24.6) |
| Cannula malposition ^b^ | 53 (4.7) | 15 (4.1) | 12 (9.3) |
| Unsuccessful cannulation ^c^ | 4 (0.4) | 2 (0.6) | 5 (3.9) |
| Cannulation-related bleeding ^d^ | 192 (17.0) | 59 (16.1) | 17 (13.2) |
| Others | 16 (1.4) | 6 (1.6) | 4 (3.1) |
| ECMO-related complications ^e^ | 38 (3.5) | 7 (1.9) | 5 (4.0) |
| Hemorrhage ^f^ | 91 (8.1) | 36 (9.8) | 11 (8.5) |
| Ischemia ^g^ | 20 (1.8) | 4 (1.1) | 2 (1.6) |

Data are presented as median [interquartile range] for continuous variables and as *N* (percentage) for categorical variables.

ECPR = extracorporeal cardiopulmonary resuscitation, ECMO = extracorporeal membrane oxygenation

^a^ Patients may have more than 1 complication.

^b^ Cannulation requiring correct the position, or cannulation of wrong vessel such as arterial-arterial and veno-veno cannulation

^c^ Failure to complete cannulation

^d^ Including cannulation site bleeding and retroperitoneal hemorrhage requiring blood transfusion or surgical intervention/interventional radiology (IVR)

^e^ Equipment malfunction, hemolysis, and other complications related to ECMO

^f^ Including intracerebral hemorrhage noted on computed tomography (CT), mediastinal hemorrhage, intraabdominal organ hemorrhage, and gastrointestinal hemorrhage requiring blood transfusion or surgical intervention/IVR

^g^ Including cerebral infarction and mesenteric ischemia

Missing data on initial cardiac rhythm at the scene = 16

**Table S6. ECMO information**

| **Variables** | **Total (N = 1,644)** |
| --- | --- |
| Cannulation technique |  |
| Percutaneous | 1,582 (98.0) |
| Surgical | 32 (2.0) |
| Others | 1 (0.06) |
| Location of cannulation |  |
| Emergency department | 1,049 (64.2) |
| Catheterization room | 581 (35.6) |
| Others | 3 (0.2) |
| Size of ECMO cannula, Fr |  |
| Arterial | 16.0 [15.0–16.5] |
| Venous | 21.0 [19.5–21.0] |
| Distal limb perfusion | 384 (23.9) |
| Before ischemic symptoms | 294 (78.2) |
| After ischemic symptoms | 81 (21.5) |
| Duration of ECMO among survivors, days | 4 [3–5] |
| Removal method of cannula removal among survivors |  |
| Compression | 170 (40.7) |
| Surgical | 248 (59.3) |

Data are presented as median [interquartile range] for continuous variables and as *N* (percentage) for categorical variables.

ECMO = extracorporeal membrane oxygenation

Missing data: cannulation technique = 29, location of cannulation = 11, size of arterial ECMO cannula = 158, size of venous ECMO cannula = 159, distal limb perfusion = 35, time of distal limb perfusion = 8, duration of ECMO among survivors = 22, method of cannula removal among survivors = 29
